# Self-esteem in patients with venous thromboembolism predicts time trade-off values for own health

**DOI:** 10.1186/s12955-022-01947-5

**Published:** 2022-03-05

**Authors:** Peep F. M. Stalmeier, Eva E. Volmeijer

**Affiliations:** 1 Radboud Institute for Health Sciences, Radboud University Nijmegen Medical Centre, HEV 138, P.O. Box 9101, 6500 HB Nijmegen, The Netherlands; 2 Department of Research and Innovation, Sint Maartens Clinic, Nijmegen, The Netherlands

**Keywords:** Self-esteem, Time trade-off, Health related quality of life, Health status, Medical decision making, Health economics, Venous thromboembolism, Hypothetical health states, Current health, Self-experienced health, C44, D71, I18

## Abstract

**Background:**

The time trade-off (TTO) is a commonly used preference based method to assess health related values used in health economic analyses. Surprisingly little is known about the factors influencing the TTO. Since self-esteem is a predictor for health status measurements, and health status affects the TTO, we studied the relation between self-esteem and TTO values.

**Methods:**

Data of 128 patients treated with vitamin K antagonists for venous thromboembolism on Short Form-36 (SF-36), Rosenberg self-esteem and patient characteristics were collected. TTO values were obtained for ‘current health’ and three chronic health states related to thrombosis, in face-to-face interviews with patients. Regression analyses were performed with the TTO as dependent variable. Analyses were performed in two groups; the complete sample, and traders only. Selected predictors were entered in four blocks: socio-demographic factors, medical-clinical factors, health status, and self-esteem.

**Results:**

In the complete sample (N = 128), bivariate regression analysis showed that self-esteem explained 14% of the variance in TTO values for current health (*p* < .000, N = 117). In traders, multivariate regression analysis showed a significant relationship between self-esteem and TTO values for current health. Self-esteem increased the variance explained (R^2^) by 8.8%, from 28.1 to 36.9%, (*p* = 0.01; N = 57). For hypothetical health states, the effect of self-esteem was weaker and mostly absent after controlling for selected variables.

**Conclusions:**

In patients willing to trade-off time, higher self-esteem was associated with higher TTO values for own current health. Self-esteem explained an appreciable proportion of the variance in TTO values in traders. For hypothetical health states such associations were weak or absent.

## Introduction

Preference-based instruments express the value or desirability of health in a single score [1]. Commonly used preference-based instruments are the standard gamble, time trade-off (TTO), rating scale and discrete choice experiments [1–4] {Arons, 2013 #79}. The TTO is a widely used health valuation measure in a large variety of countries. It has been used to obtain tariffs for health economic decision making. The TTO involves individual judgements of health relative to death and perfect health. Respondents sacrifice hypothetical life years in good health to avoid living in a lesser health state [1]. Sacrificing more life years would be expected for more severe health states, and corresponds with lower TTO values. In general, respondents may value their own health, that is self-experienced health, or hypothetical health states.

Despite its popularity, surprisingly little is known about factors influencing TTO values. In other words, the construct validity of the TTO, that is whether and to what extent preferences for health differ according to characteristics of respondents, has not been systematically studied [5]. Such knowledge is important to interpret TTO values, e.g. how the TTO will function in various contexts. It is true that medical factors have been shown to affect TTO or TTO based tariffs [6–13]. However, for other patient characteristics, such as socioeconomic variables, only weak associations with TTO values were reported [14–18]. It was also found that such relations were stronger for patients willing to trade time [17, 19, 20].

This study examined self-esteem as a TTO-determinant for three reasons. First, self-esteem is a positive predictor of several health status measures [21–26]. As only weak positive relations between health status measures and the TTO have been reported, it is not evident that self-esteem affects TTO values [19, 27–30]. Nevertheless, a positive relation was expected. Second, this study tests Tsevat’s hypothesis that self-esteem is positively related to the TTO. His hypothesis was based on qualitative observations in patients infected with human immunodeficiency virus (HIV), reporting TTO values for their self-experienced health [31, 32]. Third, the TTO confronts patients with ‘dead’. A sociopsychological theory relevant to death thoughts is Terror Management Theory [31, 33, 34]. The theory implies that “individuals’ valuations change when confronted with death in such a way that the standards and values, through which the individual acquires meaning in life and value in him/herself, are strengthened” [35, 36]. Self-esteem figures strongly in Terror Management Theory and it was conjectured to be positively related to TTO values and behavior [35, 36].

Taking into account these considerations, our research questions are: in patients who experienced venous thromboembolism, (1) is self-esteem related to the TTO values for their own health and hypothetical health, and (2) what is the relation between TTO and self-esteem in the subgroup of patients willing to trade life years.

## Methods

### Patients

Three groups of eligible patients were recruited between October 2000 and June 2002: (a) newly diagnosed patients with a first or second episode of venous thromboembolism for whom treatment with vitamin K antagonists had been started; (b) patients who had experienced an episode of major bleeding during treatment with vitamin K antagonists in the previous year; (c) patients with a post-thrombotic syndrome diagnosed at least one year after an episode of deep vein thrombosis, who had been treated with vitamin K antagonist for at least three months. Excluded were patients younger than 18 years, patients with a cancer or other serious co-morbidity, as well as patients with an insufficient knowledge of the Dutch language [12, 37].

The present study was performed at three academic centres: The Academic Medical Centre in Amsterdam, the University Hospital of Groningen, and the University Hospital of Maastricht. In each center, the study had been approved by the institutional review board [12].

### Procedures

Eligible patients were invited to join the study by a physician or research nurse who explained the purpose of the study and provided written information [12]. Written informed consent was obtained. Physicians gathered medical-clinical data during the consultation by means of the medical dossier and by asking their patients directly. Afterwards patients were handed a questionnaire, assessing quality of life with the SF-36 instrument, self-esteem, and socio-demographic variables. They were asked to return this questionnaire by mail within 2 weeks. Also, arrangements were made for an interview in which the TTO assessment was included. As we wanted patients to have experienced treatment with vitamin K antagonists at the time of the interview, patients with an episode of venous thromboembolism were interviewed approximately three months after inclusion. Patients who experienced a major bleeding event and patients with a post-thrombotic syndrome had already been treated with vitamin K antagonist, and were therefore contacted a few days after inclusion to schedule an interview appointment in the near future.

All patients were interviewed by one of four trained interviewers. For quality control, each interviewer audio taped three of the first ten interviews and received detailed feedback from the investigators. On average, the interview lasted 62 min (range 32–120) [12].

### Measures

#### Socio-demographic variables

In the questionnaire, information was gathered regarding patients’ sex, age, marital status, employment status, education, religion and family history of thromboembolism.

#### Time trade-off (interview)

TTO questions were asked face to face for eight thrombosis related health states and ‘current health’ [12]. Health states were described by vignettes mentioning concomitant physical, social, and mental domains. Vignettes were developed by clinical experts in the field. The vignette for ‘current health’ simply asked patients to consider these three domains for the past week while valuing their own health. Life expectancies, to be used in the TTO, were determined using tables from the CBS-statistics in the Netherlands [38]. Five health states were temporary and not considered here. Here, we study ‘current health’, and three chronic health states namely ‘non-fatal haemorrhagic stroke’, ‘post-thrombotic symptoms’, and ‘no treatment with vitamin K antagonists’ [12]. The TTO question asked the patient to choose, using a series of choices, between the health state under consideration for their remaining life expectancy (*t*), and a variable amount of time in perfect health (*x*); both states are followed by death [37]. In the first two choices, the duration of time in perfect health was set equal to zero or remaining life expectancy, in random order. The bisection method was used to vary the time spent in perfect health until the patient reported indifference between the two options. The value for own current health was calculated by dividing *x* by *t* and multiplying it by 100. The TTO therefore ranged from 0 (representing death) and 100 (representing perfect health) [12, 37]. The research team decided not to elicit values worse than dead for ethical reasons, and because of the complicated nature of such questions.

#### Rosenberg self-esteem (questionnaire)

The Rosenberg self-esteem instrument assesses patients’ self-esteem using a questionnaire in which ten items are rated on a four-point Likert scale ranging from ‘strongly agree’ to ‘strongly disagree’. Examples of items are: “I feel that I’m a person of worth, at least on an equal plane with others” and “At times I think I am no good at all”. The scale was computed by rescaling the negative questions and then adding the ten scores. Scores were only computed when all ten items were answered. The total Self-esteem score ranges from 10 to 40, with higher values indicating higher self-esteem [39].

#### Short form health survey (SF-36 Questionnaire)

The SF-36 health survey is a standardized questionnaire used to assess patient health status across eight dimensions [40]. There is extensive evidence of the ability of these scores to describe the health differences between different patient groups and, more important for evaluation, their ability to detect health changes following intervention [41–44]. The SF-36 instrument measures eight domains of health: physical functioning, role limitations due to physical problems, bodily pain, general health perceptions, vitality, social functioning, role limitations due to emotional problems, and mental health. The validated Dutch version of the Medical Outcomes Study 36 item Short Form Health Survey (SF-36) was used [45]. The timeframe employed was the previous week. Each of the eight domains was computed if at least 50% of the questions in that category were answered. All scores were linearly converted to a 0–100 scale, with higher scores indicating better levels of health status [46, 47]. The Mental Component Summary score (MCS) and Physical Component Summary score (PCS) were computed following a standardized three step procedure [46, 47]. The mean and standard deviation of the U.S. population were used for transforming and computing summary scores [46, 47]. Patients’ SF-36 scores were compared with published age and sex-matched Dutch reference population norms that were computed identically [45].

### Statistical analysis

All analyses were performed using SPSS 25.0 for windows. Prior to analysis we examined patient characteristics and primary outcome variables. In our study we sought to establish whether self-esteem is a predictor for TTO values. We performed statistical analyses in two selections of patients: (1) analyses in the complete sample, and (2) analyses in traders. Traders are respondents willing to trade-off time in the Time Trade-off task to gain better health. Non-traders are not willing to trade-off any time to gain better health, so their utility equals 1.

In general, non-traders should be included in the sample to estimate mean TTO values. However, our research question is about associations. As all TTO values in non-traders are equal to 100, whatever the value of the predictor, correlations of any predictor with the TTO value will be zero. Thus, larger proportions of non-traders in the sample will weaken or mask possible relations between predictors and the TTO [17, 19, 20]. For this reason, separate analyses in non-traders were done.

In the *complete* and traders’ samples, we examined bivariate relationship between self-esteem and TTO. For multivariate analyses, several blocks of variables were analyzed to select possible predictors. The blocks consisted of socio-demographic and medical-clinical factors, and the SF-36 summary scales. Each block of potential predictors was entered separately in multivariate regression analysis; predictors with *p* < 0.2 were selected for our final multivariate models. In the final multivariate models, a result with a *p* value lower than 0.05 was considered significant.

## Results

The sample comprised 128 respondents of which 3 (2.3%) had missing TTO values and 46 (35.9%) were not willing to trade-off any time. Table 1 shows the patient characteristics of our study population: 46.8% was male with a mean age of 52.5 (sd = 15.64), 61 (54%) had paid work, 59 (48.4%) were religious, 83 (66.9%) were married or living together, and 59 (50.4%) had no comorbidities. Patient characteristics were comparable with the Dutch population [45]. A summary description of the main variables (TTO, MCS, PCS and self-esteem) is shown in Table 2. Respondents on average scored 48.61 (sd = 12.07) on the MCS scale and 39.64 (sd = 10.76) on the PCS scale. These scores were substantially lower than the scores for the MCS 52.1 (sd = 9.6) and the PCS 49.7 (sd = 9.3) in the Dutch reference population [20]. Mean self-esteem was 31.08 (sd = 5.50) and ranged from 15 to 40.

**Table 1:** Patient characteristics of complete sample and traders only

	Complete sample(N = 128)	Traders (N = 79)
Gender, N (%)		
Male	58 (46.8)	33 (43.4)
Female	66 (53.2)	43 (56.6)
Marital status, N (%)		
Married or living together	83 (66.9)	48 (63.2)
Not living together	41 (33.1)	28 (36.8)
Highest finished education, N (%)		
Low	46 (37.1)	27 (35.5)
Middle	52 (41.9)	33 (43.4)
High	26 (21.0)	16 (21.1)
Paid work, N (%)		
Yes	61 (50.4)	40 (54.1)
No	60 (49.6)	34 (45.9)
Religious, N (%)		
Yes	59 (48.4)	38 (50.7)
No	63 (51.6)	37 (49.3)
Mean age, (sd)	52.52 (15.64)	53.00 (15.27)
Sum of comorbidities, N (%)^a^		
0	59 (50.4)	32 (44.4)
1–2	53 (45.3)	36 (50.0)
3–4	5 (4.3)	4 (5.6)
Pulmonary embolism, N (%)		
First	12 (9.4)	7 (9.0)
Second	3 (2.4)	2 (2.6)
None	112 (88.2)	69 (88.5)
Deep venous thrombosis, N(%)		
First	29 (22.8)	19 (24.4)
Second	12 (9.5)	5 (6.4)
None	86 (67.7)	54 (69.2)

Some respondents had missing values on one or more variables; proportions given are valid proportions rounded to sum up to 100%

^a^The five comorbidities included in this variable are: diabetes mellitus, hypertension, cardiovascular diseases, pulmonary diseases and ‘else’

**Table 2:** Description of primary outcome variables of total study population and traders only

	Complete sample		Traders only	
	Mean (sd)	Median (min–max)	Mean (sd)	Median (min–max)
TTO	88.61 (17.19)	98.75 (16.67–100)	81.98 (19.18)	89.58 (16.67–99.72)
MCS	48.61 (12.07)	52.06 (18.72–69.98)	46.93 (12.36)	50.04 (22.96–68.71)
PCS	39.64 (10.76)	38.65 (19.13–61.20)	37.73 (10.41)	35.94 (19.13–57.53)
Self-esteem	31.64 (5.27)	31.00 (15.00–40.00)	31.08 (5.50)	31.00 (15.00–40.00)

TTO, time trade-off; MCS, Mental Component Summary Scale; PCS, Physical Component Summary Scale;

### Results in the complete sample

In the complete sample, TTO values for ‘current health’ were negatively skewed with a mean and median value of 88.5 and 98.75 respectively, N = 124. 35.9% of the patients were non-traders. In bivariate analysis self-esteem was related to TTO values for ‘current health’ (r = 0.374, *p* < 0.000, N = 117) explaining 14% of the variance of TTO values. A multivariate model including the block-wise selected variables gender, comorbidities, and PCS, showed that adding self-esteem increased the explained variance by 7.6%, from 22.2 to 29.8%, *p* = 0.002, N = 97 (data not shown). Comorbidities was still significant in the full model at *p* = 0.003).

In the complete sample, TTO values for the hypothetical health state ‘non-fatal haemorrhagic stroke’ were strongly positively skewed with a mean and median value of 23.5 and 0, respectively, N = 123. 57% of the patients gave a value of zero (all-in traders). In bivariate analyses, self-esteem was not related to TTO values (r = 0.024, *p* = 0.79, N = 116), therefore further analyses were not considered.

In the complete sample, TTO values for the hypothetical health state ‘post-thrombotic symptoms’, were negatively skewed, with a mean and median, of 83,8, and 90,4, respectively (N = 124). 22.7% of the patients were non-traders. In bivariate analyses, self-esteem was not related to TTO values (r = 0.17, *p* = 0.07, N = 124), therefore further analyses were not considered.

In the complete sample, TTO values for the hypothetical health state ‘no treatment with vitamin K antagonists’, were negatively skewed with a mean and median value of 89.9 and 98.8, (N = 124). 39.8% of the patients were non-traders. In bivariate analyses, self-esteem was related to the TTO values for ‘no treatment with vitamin K antagonists’, (r = 0.236, *p* = 0.01, N = 117), explaining 5.7% of the variance. A multivariate model including the block-wise selected variables gender, ‘light to mild post thrombotic symptoms’ and ‘severe post thrombotic symptoms’ showed that adding self-esteem increased the explained variance explained by 5.2%, that is from 6.5% to 11.7%, *p* < 0.01, N = 117) (data not shown). The variables ‘mild to light, and ‘severe post thrombotic symptoms’ remained significant at *p* = 0.32, and *p* = 0.012 respectively.

### Results in traders

In the traders group, TTO values for ‘current health were examined’. Three respondents had missing values, leaving 74 traders for analysis. In bivariate analyses, self-esteem was related to the TTO values for ‘current health’, (r = 0.427, *p* = 0.000, N = 74), explaining 18.2% of the variance in TTO-values. Selected predictors for the restricted model were ‘mild to light post thrombotic symptoms, ‘severe post thrombotic symptoms’, comorbidities, recent events, and PCS (see Table 3). The results of the restricted model are presented in the upper part of Table 4, explaining 28.1% of the variance. There were 57 respondents left due to attrition caused by missing data. The full model (N = 57, lower part of Table 4) showed that adding self-esteem increased the explained variance by 8.8%, that is from 28.1% without self-esteem, to 36.9% including self-esteem (*p* = 0.011). Severe post thrombotic symptoms and comorbidities were still significant in the full model at *p* = 0.079, and *p* = 0.009, respectively.

**Table 3:** In traders only, four separate multivariate linear regressions to select predictors for the TTO

Predictors entered	LL 95% CI	Estimate	UL 95% CI	β	*p*
Sociodemographic variables N = 74, R^2^ = 0.09					
Constant	52.995	80.938	108.881	–	< 0.001
Gender	− 3.882	5.290	14.581	0.141	0.257
Paid work	− 4.808	6.143	16.853	0.165	0.264
Religion	− 11.556	− 2.271	6.557	− 0.073	0.551
Education	− 1.661	0.746	3.654	0.073	0.575
Living status	− 10.980	− 1.659	8.018	− 0.043	0.728
Age	− 0.460	− 0.103	0.272	− 0.084	0.576
Medical-clinical factors. N = 63, R^2^ = 0.255					
Constant	65.764	106.635	147.505	–	< 0.001
PTS: light to mild symptoms	− 0.961	1.906	4.772	0.230	0.188 #
PTS: severe symptoms	− 4.027	− 1.635	0.757	− 0.221	0.176 #
Number of comorbidities	− 10.611	− 5.924	− 1.237	− 0.329	0.014 #
Sum of symptoms	− 50.961	− 14.674	21.614	− 0.096	0.421
Recent events	− 20.574	− 9.704	1.165	− 0.213	0.079 #
Severe bleeding	− 24.590	− 9.064	6.462	− 0.158	0.247
Family history	− 8.103	− 1.143	5.816	− 0.043	0.743
Quality of life, SF-36 summary scales N = 68, R^2^ = 0.132					
Constant	37.548	58.781	80.014	–	< 0.001
PCS	0.237	0.683	1.130	0.367	0.003#
MCS	− 0.398	− 0.024	0.349	− 0.016	0.897
Rosenberg self-esteem. N = 74, R^2^ = 0.182					
Constant	9.775	34.184	58.594		0.007
Rosenberg self-esteem scale	0.781	1.555	2.329	0.427	< 0.001#

TTO, time trade-off; LL, lower limit; UL, upper limit; CI, confidence interval; β, standardized regression coefficient; PCS, Physical Component Summary Scale; PTS: light to mild symptoms, post thrombotic syndrome: sum of light to mild symptoms; PTS: severe symptoms, post thrombotic syndrome: sum of severe symptoms; recent events, number of recent events with an impact on health

^#^Selected predictors with *p* < 0.2

**Table 4:** In traders only, multivariate linear regression model with selected predictors for TTO values

	LL 95% CI	Estimate	UL 95% CI	β	*p*
Restricted model: N = 57, R^2^ = 0.281					
Constant	59.27	78.89	98.69		0.000
PTS: light to mild symptoms	− 1.16	1.728	4.61	0.218	0.236
PTS: severe symptoms	− 4.402	− 2.000	0.389	− 0.292	0.099
Recent events	− 16.79	− 6.139	4.509	− 0.141	0.253
Number of comorbidities	− 11.45	− 6.88	− 2.31	− 0.385	0.004*
PCS	− 0.137	0.333	0.803	0.186	0.161
Full model: N = 57, R^2^ = 0.369					
Constant	20.07	49.34	78.616	–	0.001
PTS: light to mild symptoms	1.031	1.705	4.441	0.215	0.217
PTS: severe symptoms	− 4.291	− 2.023	0.245	− 0.294	0.079
Recent events	− 12.228	− 1.564	9.101	− 0.036	0.294
Number of comorbidities	− 10.345	− 5.959	− 1.572	− 0.334	0.009*
PCS	− 0.277	0.182	0.642	0.102	0.429
Rosenberg self-esteem	0.261	1.092	1.923	0.333	0.011*

TTO, time trade-off; LL, lower limit; UL, upper limit; CI, confidence interval; β, standardized regression coefficient; PTS: light to mild symptoms, post thrombotic syndrome: sum of light to mild symptoms; PTS: severe symptoms, post thrombotic syndrome: sum of severe symptoms; recent events, number of recent events with an impact on health; PCS, Physical Component Summary Scale

*Significant *p* value

In the traders group, values for the hypothetical health state ‘non-fatal haemorrhagic stroke’ were examined. Instead of removing non-traders, all-in traders were removed because 57% of the respondents gave a value of zero. In bivariate analyses, self-esteem was unrelated related to the TTO values*.* Therefore further analyses were not considered.

In the traders group, values for the hypothetical health state ‘post-thrombotic symptoms’ were examined. In bivariate analyses, self-esteem was related to the TTO values (r = 0.211, *p* = 0.045, N = 91), Using the block wise selection procedure, only living status was selected. The explained variance in the restricted model without self-esteem was 4.6%, *p* = 0.024, N = 91). Adding self-esteem did not increase the amount of variance explained (increase = 3.7%, *p* = 0.067).

In the traders group, values for the hypothetical health state ‘no treatment with vitamin K antagonists’ were examined. In bivariate analyses, self-esteem was related to the TTO values (r = 0.281, *p* = 0.018, N = 70). Selected predictors were gender, working status, education, light and severe post thrombotic symptoms and comorbidity. The explained variance in the restricted model was 22% (N = 62). Adding self-esteem did not increase the amount of variance explained (increase = 4.5%, *p* = 0.075).

### Summary of the results

In the complete and traders samples, TTO values for current health were moderately related to self-esteem, both in bivariate as well as in multivariate analysis controlling for selected variables. In the complete sample, for 2 out of 3 hypothetical health states, bivariate relations showed that self-esteem was weakly related TTO values; for one hypothetical health states, self-esteem significantly added 5.2% of the variance after controlling for selected variables. In traders, bivariate analyses again showed that self-esteem was weakly related to TTO values for 2 out of 3 hypothetical health states. The contribution of self-esteem was no longer significant after controlling for selected variables.

## Discussion

We sought to establish whether self-esteem is a predictor of TTO values in order to investigate the construct validity of the TTO values. In our study, the sample consisted of 128 patients treated with vitamin K antagonists for venous thromboembolism. Among all respondents, adding self-esteem increased the explained variance by 7.6%. Among patients willing to trade time, after controlling for selected predictors, adding self-esteem increased the explained variance of TTO for ‘current health’ by 8.8%. For hypothetical health states, the effect of self-esteem was weaker and mostly absent after controlling for selected variables.

### Interpretation

The findings for ‘current health’ are discussed first. The physical symptoms scale of the SF-36 (PCS) was related to TTO values for ‘current health’. This is reasonable since if SF-36 scores for one’s own health improve, one expects higher value for one’s current health. The relation between PSC and TTO current health values was found in the complete sample and in traders. These results replicate the findings in other studies reporting correlations between SF-36 and TTO values ranging from 0.01 to 0.60 [19, 27–30, 48]. When self-esteem was included, a sizeable amount of additional variance was explained: 7.6% in the complete same and 8.8% in traders. Thus our study establishes that self-esteem is an independent predictor of TTO values, even after controlling for the SF-36 health status measure. Our study confirms results of Mrus who found in veterans and in bivariate analyses, that an increase in self-esteem predicted standard gambles values for ‘current health’ [39, 49]. Our study confirms an earlier hypothesis of Tsevat that self-esteem is a predictor of the TTO [31, 32]. His hypothesis was based on qualitative observations in patients infected with human immunodeficiency virus (HIV), valuing their self-experienced health whereas our quantitative results are obtained in an observational study. Our results suggests that raising self-esteem is a relevant target to address in low-self-esteem populations {Rodriguez-Ayllon, 2019 #73}{Gow, 2020 #74}{Siette, 2017 #76}.

For the hypothetical states, self-esteem did not significantly affect the TTO-values in all but one analysis, that is for the hypothetical health state ‘no treatment with vitamin K antagonists’, in the complete sample. The results suggest that the effect for hypothetical states is weaker. We offer two possible explanations. First. we expected self-esteem to be important while valuing one’s own health, but less so while valuing hypothetical health states. This was based on the assumption that valuing one’s own health generates a self-focused condition. In such a condition, thoughts occur such as ‘who am I’, or ‘what is important to me’, thoughts which touch on self-esteem [50]. We assume that such a self-focused condition is less strong in hypothetical health states, where respondents are more concerned with judging attributes and their levels. A second explanation may be a lack of power given the weak relations between self-esteem and TTO values. Post-hoc power analyses showed that the achieved power for the hypothetical health states in traders was around 52%.

### Implications for the hypothesized role of self-esteem and Terror Management Theory

TTO arguably engenders thoughts of death and Terror Management Theory is about death thoughts. Therefore, a connection between TTO values and Terror Management Theory was suspected. In Terror Management Theory, self-esteem is a core concept and an important defense against death thoughts. This was one of the reasons to include self-esteem in the current study. The relation between cultural values and TTO values is explained in detail in recent work [35]. The present results support the expected relation between self-esteem and TTO values, based on this framework [35].

### Consequences for health technology assessment agencies

To recognise the role of self-esteem in TTO values may facilitate sharing the interpretation of TTO values between the outcomes research community and healthcare decision makers, such as health technology assessment agencies. However, their decisions are usually, but not always, based on evaluations of health from the societal perspective. These are determined using societal valuations of hypothetical multi-attribute health states. For such an application, a small impact of self-esteem is expected given the weaker relations between self-esteem and TTO found here for hypothetical health states. For agencies that do indeed use values for current or self-experienced health, it is important to know that TTO values are not only affected by medical or psychological factors, but that self-esteem may play an important role.

### Directions for further research

1. In future studies, employing the TTO, the present findings can be tested in other populations by including self-esteem as a predictor.
2. In future studies, one may confirm whether the relation between self-esteem for TTO values is indeed stronger for current health as compared to hypothetical health states.
3. Terror Management Theory contains many cultural values, e.g. being patriotic, or desire for having children. Such cultural values may be tested for their association with TTO values. A large number of such values is available {Burke, 2010 #78}. Such studies may serve to strengthen the link between Terror Management Theory and TTO values.

### Limitations and strengths

A limitation is that the proportion of non-traders in our study population (35.9%) was higher than expected. The high prevalence of non-traders could be reason to question the quality of the interviews and validity of the TTO elicitation. However, the prevalence of non-traders in our sample is actually lower than the average proportion of 57% estimated in a review in other patient populations [28]. A second objection is that TTO values were skewed. However, in traders, the residuals of the regression models were normally distributed, justifying their use in traders. Another limitation is the time differences between the measurement of self-esteem at baseline and the TTO-interview, ranging from two weeks to 3 months. During this period, patients’ self-esteem status may have changed reducing possible relations. Nevertheless, despite these time differences, reliable associations were found.

A strength of this study is that the TTO values for current health were obtained in carefully conducted face to face interviews by trained interviewers. Another strength is the wide range in TTO values and self-esteem scores in facilitating the detection of possible relations.

## Conclusion

The present study provides new and relevant information on the TTO method. Traders with higher self-esteem scores gave higher TTO values for their current health, even after controlling for socio-demographic variables, health status, and medical-clinical variables. This result may seem unsurprising as self-esteem is associated with health status, albeit weakly to moderately. Therefore, it was not clear in advance that self-esteem predicts the TTO. The relation between self-esteem and TTO values for hypothetical health states were weaker or absent. In general, health status, clinical variables and demographic variables can account for only a fraction of the variance in multivariable models of the TTO [15, 19, 31]. In this light, the additional 8.8% of variance explained by self-esteem found in traders may certainly be considered sizeable.

## Data Availability

The datasets used in the current study are available from the corresponding author on reasonable request.

## Abbreviations

HRQoL: Health related quality of life
QOL: Quality of life
TTO: Time trade-off
SF-36: Medical Outcomes Study Short Form-36 Questionnaire
MCS: Mental Component Summary Scale
PCS: Physical Component Summary Scale
PTS: Post Thrombotic Syndrome
